# Supplementary material for: Spotted lanternfly predicted to establish in California by 2033 without preventative management
Source: Commun Biol. 2022 Jun 8;5:558. doi: 10.1038/s42003-022-03447-0 (PMC9177847; doi:10.1038/s42003-022-03447-0)
Supplement: Supplementary file 2 — Supplementary Information [file 42003_2022_3447_MOESM2_ESM.pdf]

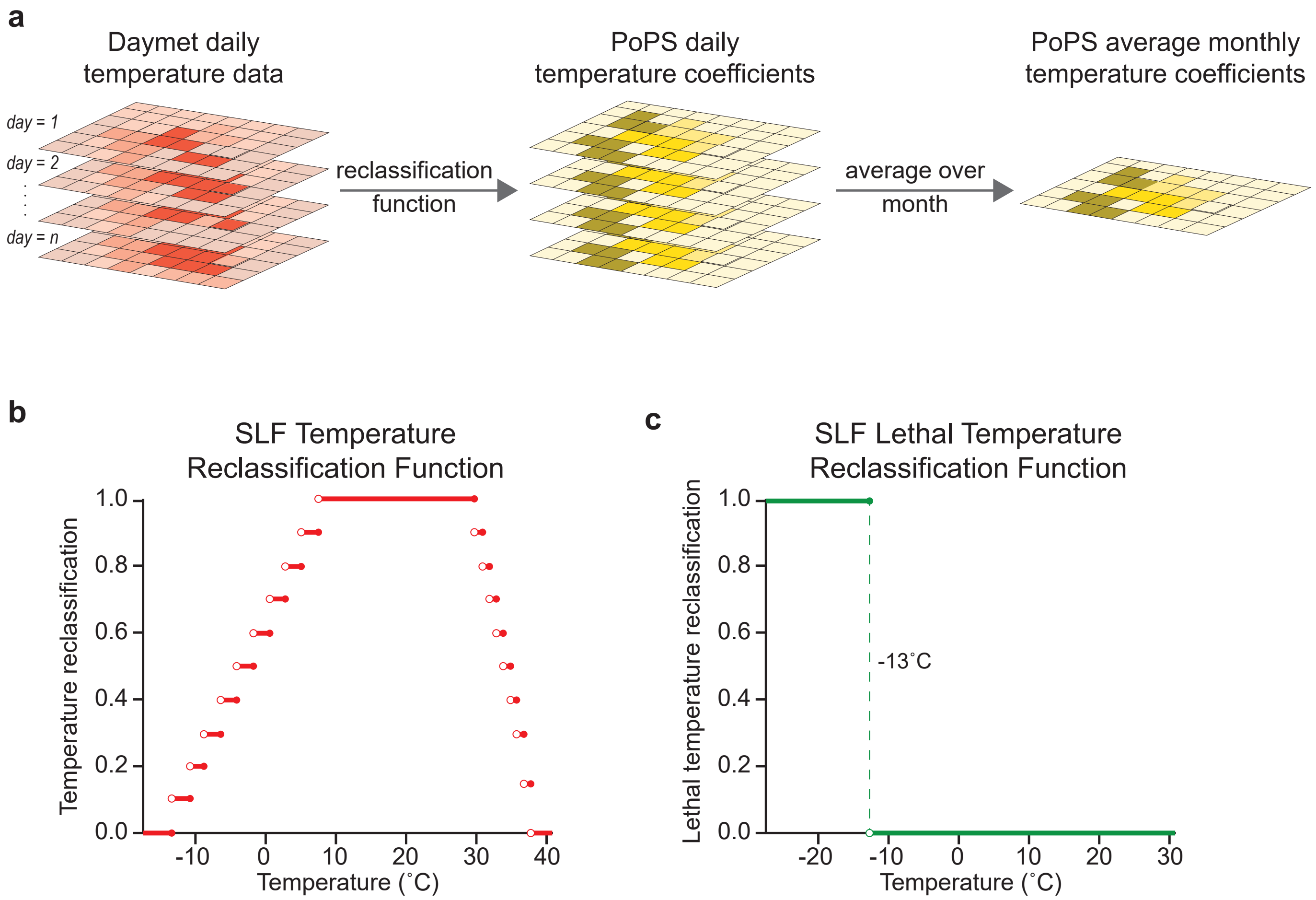

Supplementary Figure 1. Temperature reclassification. (a) All raw temperatures are converted with a reclassification function to indices ranging 0–1 to describe the impact on a species' ability to survive and reproduce. For SLF, daily Daymet temperature (b) was converted into a monthly coefficient ranging 0–1 (based on conversations with field operations personnel), and mean January temperature (c) was converted to a binary value: a value of 1 was assigned to pixels with mean daily minimum January temperature < -13º C, which is likely unsuitable for SLF overwintering.
