# Supplementary material for: Spotted lanternfly predicted to establish in California by 2033 without preventative management
Source: Commun Biol. 2022 Jun 8;5:558. doi: 10.1038/s42003-022-03447-0 (PMC9177847; doi:10.1038/s42003-022-03447-0)
Supplement: Supplementary file 3 — Description of Additional Supplementary Files [file 42003_2022_3447_MOESM3_ESM.pdf]

Description of Additional Supplementary Files

File name: Supplementary Movie 1
Description: Probability over time based on mean cell value.

File name: Supplementary Movie 2
Description: Probability over time based on maximum cell value.
