# Supplementary figures and images for: Spotted lanternfly predicted to establish in California by 2033 without preventative management
Source: Commun Biol. 2022 Jun 8;5:558. doi: 10.1038/s42003-022-03447-0 (PMC9177847; doi:10.1038/s42003-022-03447-0)

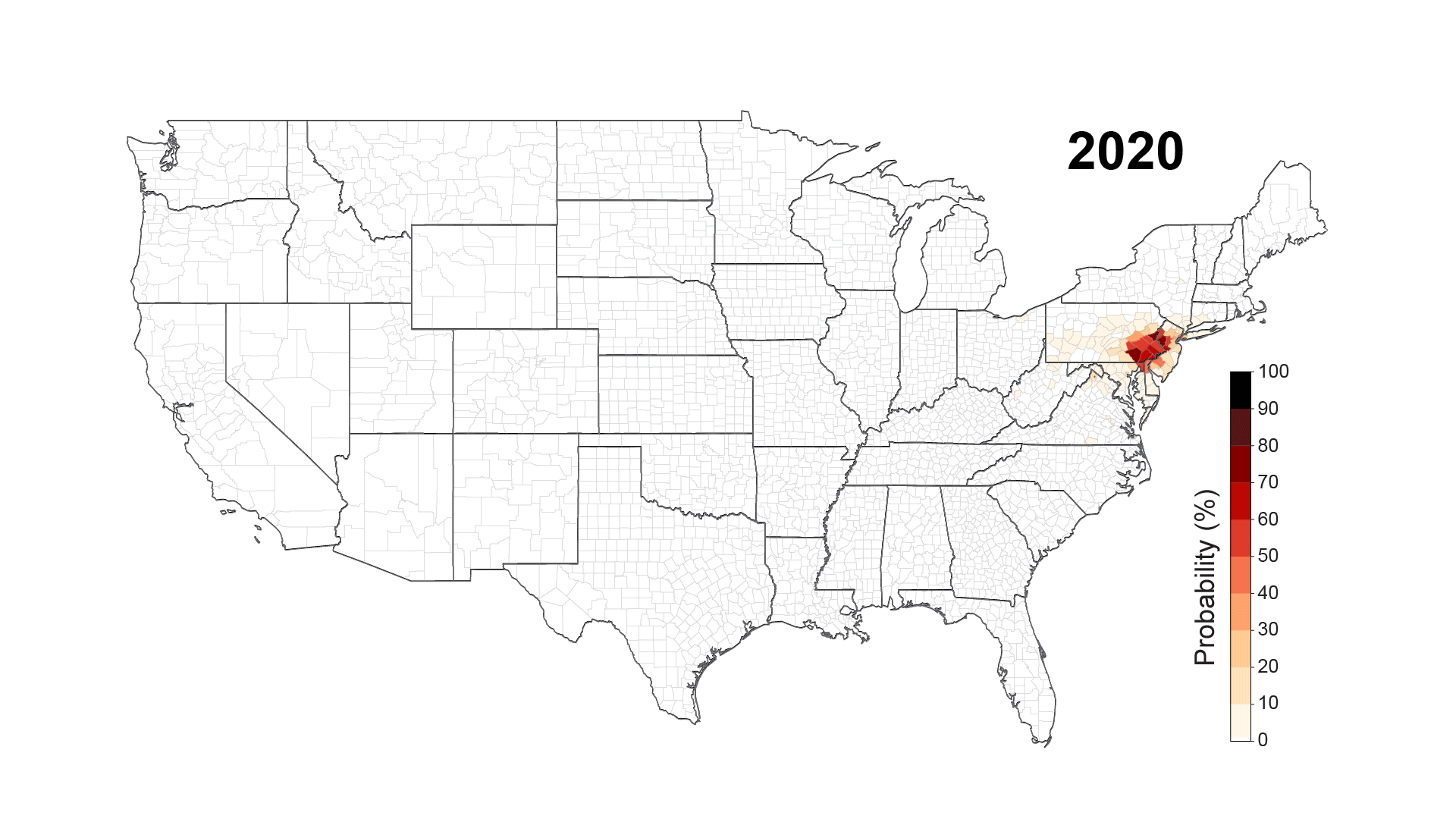

Supplement: Supplementary file 4 — Supplementary Movie 1. Probability over time based on mean cell value. [file 42003_2022_3447_MOESM4_ESM.gif]

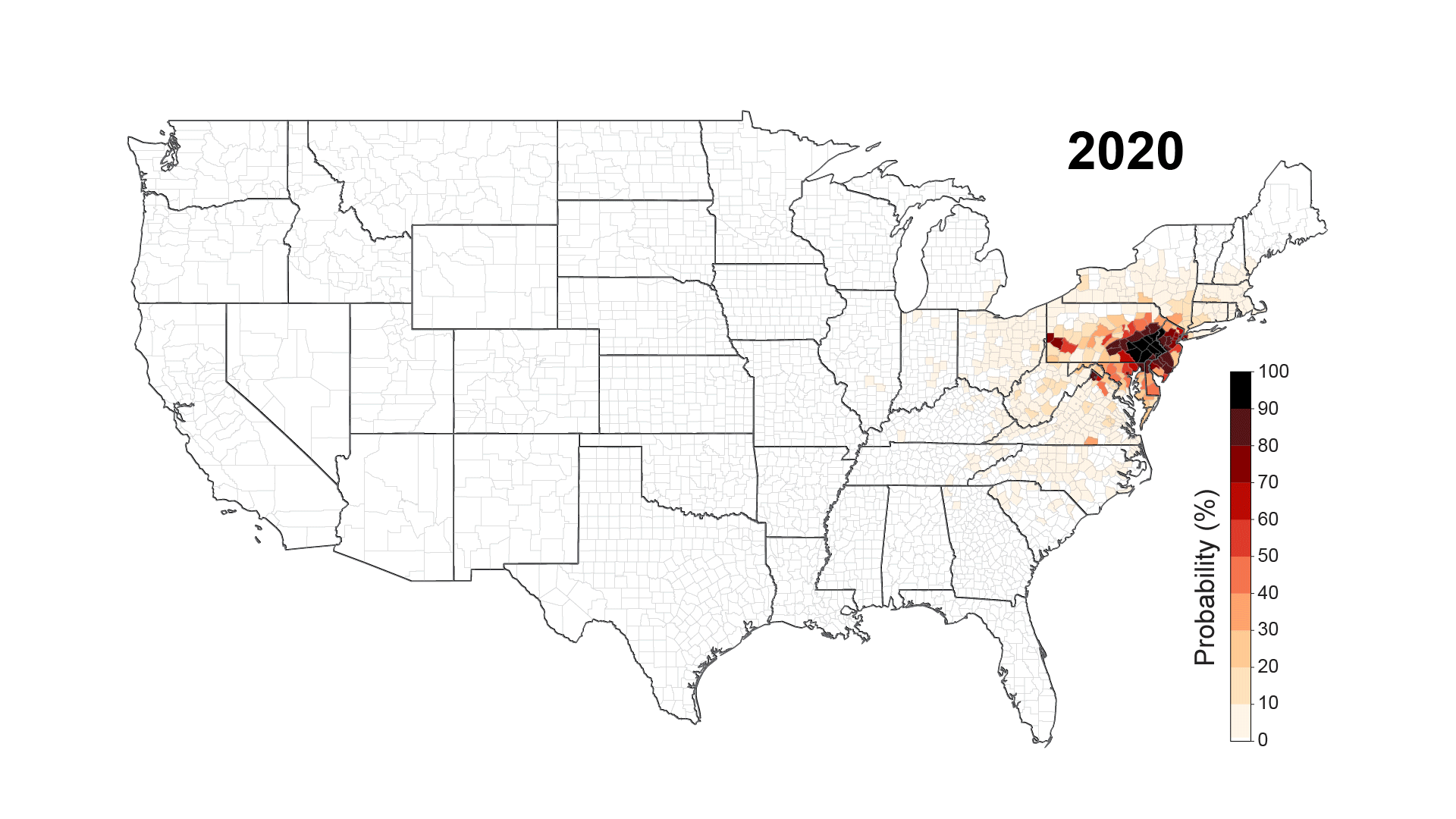

Supplement: Supplementary file 5 — Supplementary Movie 2. Probability over time based on maximum cell value. [file 42003_2022_3447_MOESM5_ESM.gif]
